# nf-core/pacsomatic: a scalable somatic analytic pipeline using PacBio HiFi data

**DOI:** 10.1093/bioinformatics/btag647

**Published:** 2026-08-27

**Authors:** Wenchao Zhang, Haidong Yi, Beifang Niu, Gang Wu, Ti-Cheng Chang

**Affiliations:** Center for Applied Bioinformatics, St. Jude Children’s Research Hospital, Memphis, TN 38105, United States; Center for Applied Bioinformatics, St. Jude Children’s Research Hospital, Memphis, TN 38105, United States; Center for Applied Bioinformatics, St. Jude Children’s Research Hospital, Memphis, TN 38105, United States; Center for Applied Bioinformatics, St. Jude Children’s Research Hospital, Memphis, TN 38105, United States; Center for Applied Bioinformatics, St. Jude Children’s Research Hospital, Memphis, TN 38105, United States

## Abstract

**Motivation:**

Pacific Biosciences (PacBio) HiFi long-read sequencing enables robust characterization of complex genomic regions, repetitive elements, and structural variants (SVs) that are often inaccessible to short-read technologies. To fully leverage HiFi reads to advance cancer genomics and epigenetics, researchers require an end-to-end, scalable and optimized bioinformatics workflow. The nf-core framework meets this need by providing rigorously tested, community-curated pipelines that ensure reproducibility, transparency, and broad compatibility across computational environments.

**Results:**

We present nf-core/pacsomatic, an automated Nextflow DSL2 pipeline designed for comprehensive paired tumor–normal somatic analysis using PacBio HiFi data. The workflow includes steps for read alignments against reference genome, somatic SNV/indel, SV, and CNV calling, CpG methylation profiling and differential methylation region (DMR) detection. Additional downstream modules support functional annotation, mutational signature analysis, tumor purity and ploidy estimation, and homologous recombination deficiency (HRD) assessment. Utilizing nf-core’s modular design and containerized execution, nf-core/pacsomatic provides a stable framework for the reproducible discovery of biological insights.

**Availability:**

nf-core/pacsomatic is available under the MIT License at nf-core (https://nf-co.re/pacsomatic) and github (https://github.com/nf-core/pacsomatic)

## 1 Introduction

PacBio long-read sequencing, a leading Third Generation Sequencing (TGS) technology, offers substantial advantages over short-read platforms by generating kilobase-scale reads capable of spanning repetitive elements and resolving complex structural variants (SVs). Its HiFi reads—derived from circular consensus sequencing (CCS)—provide a unique combination of long-range contiguity and base accuracy (>99%), establishing a foundation for high-resolution genomic analyses of both germline and somatic variation (Wenger *et al.* 2019).

The role of somatic mutations in driving cancer development is predominant, but these variants remain significantly less characterized than their germline counterparts (Milholland *et al.* 2017, Qing *et al.* 2020). Identifying somatic alterations requires specialized analytical strategies because they arise within specific cell lineages, often hide at sub-clonal levels with lower allele frequencies, and accrue over time. This complexity is mirrored in the aging process, where the accumulation of somatic mutations and concurrent epigenetic shifts serve as the primary drivers for increased cancer incidence (Qing *et al.* 2020). To truly decode tumor evolution, we need to prioritize the robust detection and characterization of the somatic genome.

Bioinformatics workflow engines such as WDL and Nextflow support the development of reproducible analysis pipelines, but they differ in design philosophy and execution behavior. WDL provides a declarative, human readable syntax and is commonly executed using engines such as Cromwell. Nextflow, by contrast, offers a powerful dataflow programming model, a Groovy-based domain-specific language, builtin parallelization, strong error handling, and seamless portability across high performance computing (HPC) clusters and cloud platforms. Although PacBio’s official longread workflows—HiFi-human-WGS-WDL (https://github.com/PacificBiosciences/HiFi-human-WGS-WDL) and HiFi-somatic-WDL (https://github.com/PacificBiosciences/HiFi-somatic-WDL)—are implemented in WDL, their execution through miniwdl limits optimization on HPC systems lacking SLURM schedulers and restricts straightforward deployment in cloud environments.

The nf-core framework provides a standardized, community-curated ecosystem in which all dependencies are encapsulated via Docker, Singularity/Apptainer, BioContainers, or Conda environments (da Veiga Leprevost *et al.* 2017, Di Tommaso *et al.* 2017, Ewels *et al.* 2020). Its rigorous testing, templates, and module library collectively ensure reproducibility, transparency, and long-term sustainability. The recently released nf-core/pacvar (Jain and Clelland 2025) mirrored HiFi-human-WGS-WDL, demonstrating how PacBio long-read germline workflows can be efficiently translated from WDL to Nextflow. Similarly, we developed nf-core/pacsomatic, a workflow for PacBio HiFi–based somatic analysis. Inspired by the logic of HiFi-somatic-WDL but implemented entirely in Nextflow DSL2, nf-core/pacsomatic is maintained as an official nf-core pipeline (Ewels *et al.* 2020), receiving community-driven validation, continuous integration, and standardized documentation.

By integrating into the nf-core ecosystem, nf-core/pacsomatic delivers a portable and reproducible framework that minimizes user configuration and simplifies deployment across HPC and cloud infrastructures. The pipeline provides researchers with an end-to-end, out-of-the-box solution for long-read somatic profiling, supporting small-variant calling, structural variant detection, CpG methylation analysis, CNV inference, and multiple downstream annotation modules. Together, these capabilities position nf-core/pacsomatic as a flexible and scalable tool for enabling high-confidence tumor–normal analysis using PacBio HiFi data.

## 2 Pipeline description and implementation

### 2.1 Implementation

**Figure 1:**
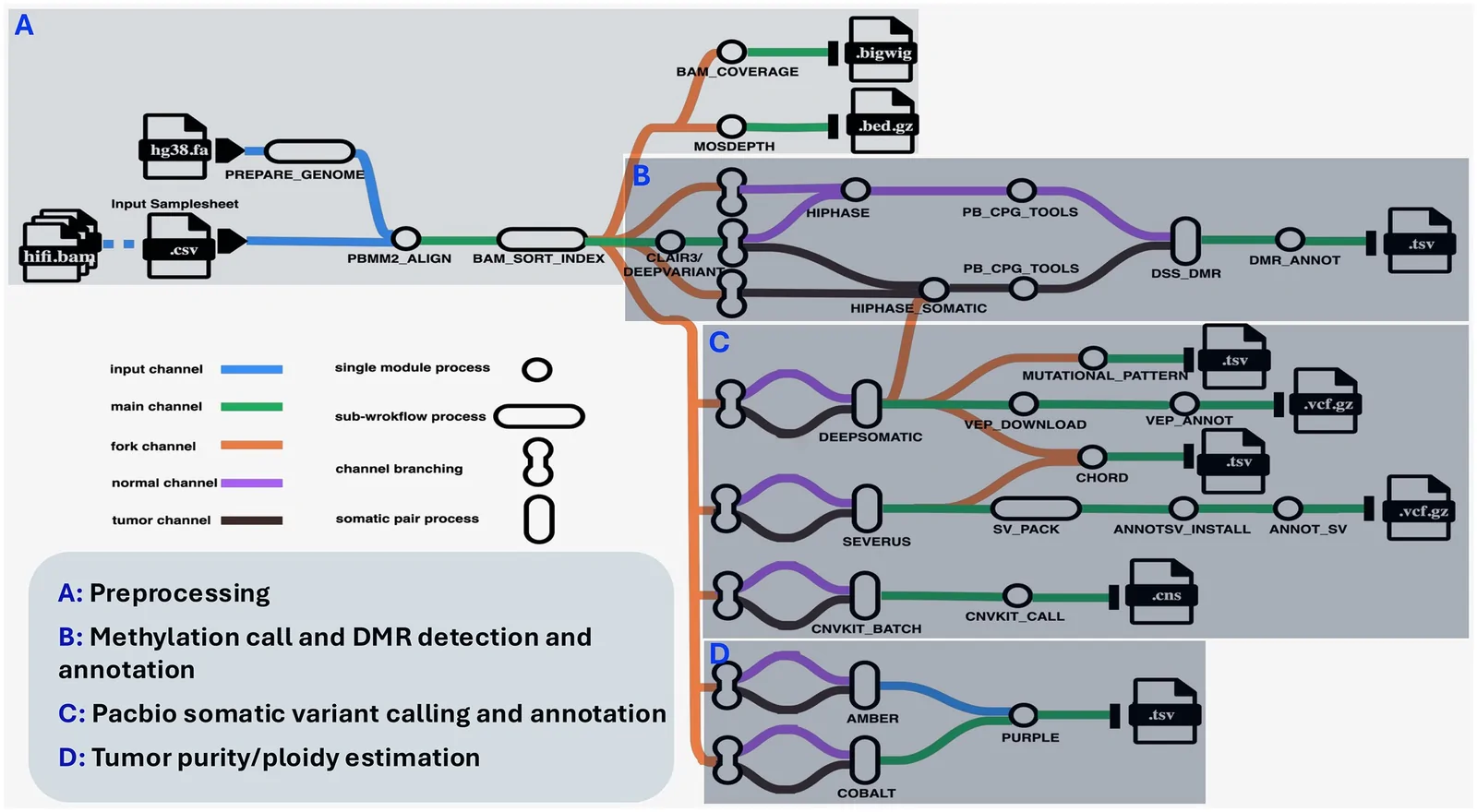
Overview of the nf-core/pacsomatic pipeline. The workflow consists of four major components: (A) preprocessing and alignment quality control (QC); (B) CpG methylation calling and differential methylation region (DMR) detection/annotation; (C) somatic SNV/indel, SV, and CNV calling with functional interpretation; and (D) tumor purity and ploidy estimation.

nf-core/pacsomatic processes PacBio HiFi tumor and normal BAM files together with a reference genome. The pipeline automatically pairs tumor–normal samples and orchestrates downstream analyses through modularized sub-workflows. As summarized in Fig. 1, the pipeline consists of four major components: (A) preprocessing and alignment quality control (QC); (B) CpG methylation calling and differential methylation region (DMR) detection/annotation; (C) somatic SNV/indel, SV, and CNV calling with functional interpretation; and (D) tumor purity and ploidy estimation.

### 2.2 Pre-processing

Required inputs include: (1) a reference genome in FASTA format, and (2) unaligned PacBio HiFi read BAM files organized via a sample sheet. nf-core/pacsomatic automatically generates genome reference index and sequence dictionary files through the PREPARE-GENOME sub-workflow. Pacbio HiFi reads are aligned using pbmm2 (a PacBio wrapper for minimap2 (Li 2018)), followed by sorting and indexing using SAMtools (Li *et al.* 2009). To ensure data integrity, the pipeline integrates multiple QC steps. Standard SAMtools statistics (e.g. stats, flagstat) provide alignment summaries, while bedtools- bam-coverage (Ramírez *et al.* 2014) and mosdepth (Pedersen and Quinlan 2018) generate detailed coverage metrics in bedgraph, bigWig, and bed formats. Together, these tools deliver a comprehensive profile of genome coverage and long-read alignment quality.

### 2.3 Methylation calling, DMR detection, and annotation

DNA methylation is an epigenetic modification, and disruption of DNA methylation control mechanisms may lead to a variety of diseases, including cancer (Di Ruscio *et al.* 2016, Nishiyama and Nakanishi 2021). PacBio HiFi sequencing enables direct detection of CpG methylation without bisulfite conversions by measuring the polymerase kinetics. The methylation probability can be estimated and tagged by a machine-learning–based methods such as Primrose (https://github.com/mattoslmp/primrose) or Jasmine (https://github.com/PacificBiosciences/jasmine), and then preserved in HiFi reads during alignment, which enable the downstream methylation analysis.

Accurate CpG methylation calling requires germline SNV calling and haplotype phasing. In nf-core/pacsomatic, users can select either Clair3 (Zheng *et al.* 2022) or DeepVariant (Poplin *et al.* 2018) for the step of germline variant calling through the pipeline configuration. HiPhase (Holt *et al.* 2024) subsequently performs haplotype phasing and generates haplotype-phased BAM files. These BAMs are subsequently processed by pb-CpG-tools (https://github.com/PacificBiosciences/pb-CpG-tools) to compute site-level and haplotype-specific methylation scores (outputs .bed and .bw files).

Tumor–normal methylation profiles are compared using DSS (Feng and Wu 2019) to identify differentially methylated regions, and results are functionally annotated with annotatr (https://bioconductor.org/packages/release/bioc/html/annotatr.html). Importantly, because tumor and germline samples may originate from distinct tissues, DMR interpretation must consider tissue-specific methylation patterns and cellular heterogeneity to avoid confounded biological conclusions.

### 2.4 Pacbio somatic variant calling and annotation

The core analytical module of nf-core/pacsomatic performs somatic SNV, indel, SV, and CNV detection using tumor–normal pairs (Fig. 1). For small variants, nf-core/pacsomatic uses DeepSomatic (Park *et al.* 2025), a deep-learning extension of DeepVariant tailored for long-read somatic variant discovery. Resulting variants are annotated using the Ensembl Variant Effect Predictor (VEP) (Hunt *et al.* 2022) and can be further analyzed for mutational signatures via MutationalPatterns (Manders *et al.* 2022).

PacBio HiFi reads excel at resolving structural variants, including breakpoints in repetitive or complex regions that short reads often miss (Cosenza *et al.* 2022, Gao *et al.* 2025). For somatic SV detection, nf-core/pacsomatic employs Severus (Keskus *et al.* 2024), with downstream filtering using SV_Pack (https://github.com/PacificBiosciences/svpack) and functional annotation via AnnotSV (Geoffroy *et al.* 2018). Furthermore, nf-core/pacsomatic support to use CHORD (Nguyen *et al.* 2020) to assess homologous recombination deficiency (HRD), which take the SNVs and SVs as inputs.

To assess gene dosage alterations, nf-core/pacsomatic incorporates a dedicated CNV workflow based on CNVkit (Talevich *et al.* 2016). Specifically, CNVKit_Batch performs normalization and segmentation, and CNVKit_Call generates integer copy-number predictions, enabling high-resolution detection of deletions, duplications, and broader CNV patterns relevant to cancer evolution (Shlien and Malkin 2009).

### 2.5 Tumor purity/ploidy estimation

Accurate interpretation of somatic variants requires reliable estimates of tumor purity and ploidy (Luo *et al.* 2018). nf-core/pacsomatic integrates PURPLE of HMFtools (https://github.com/hartwigmedical/hmftools) to estimate tumor purity and ploidy by combining B-allele frequency (BAF) from AMBER, and read depth ratios from COBALT. To optimize performance in HPC and cloud settings, the pipeline parallelizes the AMBER and COBALT steps, significantly enhancing computational efficiency.

### 2.6 Testing and output reporting

nf-core/pacsomatic is available at https://nf-co.re/pacsomatic, with comprehensive documentation and automated unit tests following nf-core standards. Users can perform a quick validation using a provided tumor–normal test dataset and built-in execution profiles. A complete description of all output files is available at: https://nf-co.re/pacsomatic/dev/docs/output

The workflow is highly scalable—CPU and memory allocations for each module can be configured through standard Nextflow profiles, enabling efficient deployment on both HPC and cloud infrastructures.

### 2.7 Benchmark of pipeline performance

To evaluate nf-core/pacsomatic, we benchmarked the pipeline using the GIAB HG008 pancreatic cancer dataset (McDaniel *et al.* 2025), utilizing both PacBio HiFi and Illumina short-read data. For small variants (SNVs/INDELs), the pipeline demonstrated high accuracy against a five-caller short-read consensus, achieving an overall F1-score of 93.68% (95.6% recall; 91.83% precision, Supplementary Table S1, available as supplementary data at *Bioinformatics* online). It is important to emphasize that the putative false positives identified here may actually represent true biological events. Given the higher alignment accuracy in complex regions, long-read sequencing often provides a more precise characterization of variant positioning and classification than short-read alternatives (Fig. S1, available as supplementary data at *Bioinformatics* online). Structural variant (SV) performance, validated against the GIAB long-read reference, varied by class: duplications and deletions showed the stronger concordance with F1-scores of 77.7% and 66.3%, respectively (Supplementary Table 2; Supplementary Fig. 2, available as supplementary data at *Bioinformatics* online).

We further expanded our benchmarking analysis to include the SEQC somatic reference dataset (Fang *et al.* 2021), using the paired human basal-like breast cancer cell line dataset HCC1395/HCC1395-BL. DeepSomatic, as deployed in our pipeline, achieved an overall F1-score of 94.11% (93.99% recall and 94.24% precision) (Table S3; Fig. S3, available as supplementary data at *Bioinformatics* online).

In addition to somatic variant benchmarking, we used the HCC1395 dataset to evaluate the germline variant callers Clair3 and DeepVariant. Germline variants identified in both the normal sample (HCC1395-BL) and the tumor sample (HCC1395) were evaluated, and the shared and caller-specific variants from each method were compared. Our analyses indicate that the germline variants called by DeepVariant and Clair3 are comparable at the high-confidence regions (Table S4; Fig. S7, available as supplementary data at *Bioinformatics* online).

We also benchmarked haplotype-resolved methylation analyses by comparing the differentially methylated regions (DMRs) generated using germline variants called by either Clair3 or DeepVariant. The comparison included both DMR concordance (chromosomal coordinates and boundaries) and consistency of methylation levels (Fig. S8, available as supplementary data at *Bioinformatics* online). A Jaccard index of 0.983, together with the near-perfect linear relationship (spearman -value = 0.9999) between methylation values, indicates that the impact of using Clair3 versus DeepVariant on methylation analyses can be negligible.

Computational performance and resource utilization—including CPU, memory, and job duration—were tracked across all pipeline modules (Fig. S9, available as supplementary data at *Bioinformatics* online). The most resource-intensive steps were pbmm2 alignment, Clair3, and particularly DeepSomatic. To alleviate computation time, nf-core/pacsomatic allows users to configure these computationally intensive modules (e.g. Clair3, DeepVariant and DeepSomatic) to run on dedicated GPU nodes, providing several-fold acceleration.

## 3 Conclusion and future direction

nf-core/pacsomatic provides a robust solution for comprehensive somatic analysis of PacBio HiFi tumor–normal datasets. Implemented in Nextflow DSL2 and supported by the nf-core framework, the pipeline support flexible configuration. Users may execute the whole workflow end to end or selectively run specific modules depending on analytical needs.

The landscape of long-read somatic variant calling is rapidly evolving, with emerging tools such as ClairS (Zheng *et al.* 2023) and ClairS-TO (Chen *et al.* 2025) for somatic SNV discovery and SAVANA (Elrick *et al.* 2025) for long-read SV detection. As the field advances, we plan to expand benchmarking efforts (Aydin *et al.* 2025) and incorporate these methods as optional modules within future versions of nf-core/pacsomatic. Continued integration of new algorithms, along with community-driven updates through nf-core, will ensure that nf-core/pacsomatic remains a sustainable, reproducible, and state of the art framework for long-read somatic profiling in cancer genomics.

## Supplementary Material

btag647_Supplementary_Data

## Data Availability

There are no new data associated with this article.
