# Supplementary material for: nf-core/pacsomatic: a scalable somatic analytic pipeline using PacBio HiFi data
Source: Bioinformatics. 2026 Aug 27;42(9):btag647. doi: 10.1093/bioinformatics/btag647 (PMC13549938; doi:10.1093/bioinformatics/btag647)
Supplement: Supplementary file 1 [file btag647_supplementary_data.docx]

nf-core/pacsomatic: a scalable somatic analytic pipeline using PacBio HiFi data

Wenchao Zhang^#^, Haidong Yi^#^, Beifang Niu, Gang Wu^*^, Ti-Cheng Chang^*^

Center for Applied Bioinformatics, St. Jude Children’s Research Hospital, 262 Danny Thomas Place, Memphis, TN 38105, USA

#: These authors contribute equally; *: Corresponding authors

## **Supplementary Method**

### **Benchmarking of GIAB-HG008 dataset and pipeline computation efficiency**

Our primary benchmarking was conducted on HG008 sequencing data. We processed the PacBio HiFi reads through the nf-core/pacsomatic pipeline and processed the Illumina short reads using a consensus approach involving five independent somatic callers. Performance metrics were calculated by comparing small variant called from nf-core/pacsomatic against consensus caller. For comparison, the reference set was treated as the baseline (truth set), and evaluated nf-core/pacsomatic for recall (sensitivity), precision (positive predictive value), and F1-score (harmonic mean of recall and precision). Metrics are reported separately for single nucleotide variants (SNVs), insertions/deletions (indels), and combined variants (SNV + Indel). (**Supplementary Table 1 and Supplementary Figure 1**).

For structural variant (SV), we benchmarked the nf-core/pacsomatic called SVs against the Genome in a Bottle (GIAB) consortium's long-read reference SV callset. Concordance between nf-core/pacsomatic and the reference callsets was assessed using position-based matching with defined tolerances: SVs were considered concordant if they shared the same chromosome, SV type, and had start/end positions within 1 kb tolerance or exhibited ≥50% reciprocal overlap for non-BND variants (**Supplementary Table 2**). The concordant SVs were subsequently matched with known driver genes (**Supplementary Figure 2**).

The computation efficiency and resource requirement of nf-core/pacsomatic running HG008 tumor/normal pairs was tracked for this benchmark process (**Supplementary Figure 9**).

### **Benchmarking of HCC1395 dataset to include assessment of SEQC2 somatic ground-truth reference**

We further benchmarked the HCC1395 dataset using the SEQC2 somatic reference dataset. Following the instructions provided by the SEQC2 consortium (<https://sites.google.com/view/seqc2> ), we downloaded the latest SEQC release (v1.2.1) from <https://ftp-trace.ncbi.nlm.nih.gov/ReferenceSamples/seqc/Somatic_Mutation_WG/release/latest/>. We also obtained the corresponding PacBio Revio HiFi sequencing datasets, HCC1395-BL and HCC1395, from <https://downloads.pacbcloud.com/public/revio/2023Q2/HCC1395/>.

After generating the sample sheet, we executed nf-core/pacsomatic on the paired tumor–normal dataset. DeepSomatic and ClairS were configured with their respective models optimized for the PacBio Revio platform and were run using the same aligned tumor and normal BAM files. The resulting somatic variant call sets were benchmarked against the SEQC2 high-confidence reference variants using som.py from the hap.py toolkit, as recommended by the Global Alliance for Genomics and Health (GA4GH). Performance metrics, including recall and precision, are summarized in **Supplementary Table 3**.

To further compare the two callers, we used bedtools intersect and pysam to identify shared and caller-specific variants. The called variants between the two callers were visualized using Venn diagram (**Supplementary Figure 3**). We further characterized the called variants as TP, FP, and FN with the VAF distribution (**Supplementary Figure 4**) and INDEL length distribution (**Supplementary Figure 5**). Overall, DeepSomatic achieved slightly better performance than ClairS across the evaluated metrics.

**Benchmarking of germline caller (Clair3 and DeepVariant) and their impact on the detected differential methylation regions**

nf-core/pacsomatic allows users to select either DeepVariant or Clair3 as the germline small-variant caller. To evaluate the performance of these two callers and assess their impact on the downstream methylation analysis, we used the same HCC1395 sample sheet and executed nf-core/pacsomatic twice by setting the configuration parameter skip_deepvariant to true and false, respectively (**Figure 1; Supplementary Figure 6**).

Germline variants within GIAB high confidence region were identified for both the tumor (HCC1395) and normal (HCC1395-BL) samples using Clair3 and DeepVariant (**Supplementary Table 4).** Pairwise comparisons of the variant call sets were visualized using four Venn diagrams (**Supplementary Figure 7**).

Because haplotype-resolved methylation analysis depends on upstream germline variant calling and phasing, differences in germline variant calls may potentially influence the downstream differential methylation analysis. To evaluate this effect, the DMRs generated using Clair3- and DeepVariant-derived germline variants were compared. Concordance between the two DMR sets was assessed by comparing genomic coordinates and methylation values. The results are presented as a Venn diagram and methylation-value concordance plots (**Supplementary Figure 8).**

The comparison showed that DeepVariant and Clair3 produced consistent germline variant call sets and nearly identical methylation profiles. These results indicate that the choice of germline caller either as Clair3 or DeepVariant has negligible impact on the downstream DMR analysis in this dataset.

## **Supplementary Table 1. Comparison of small variant calls from the nf-core/pacsomatic vs. the consensus calls from short reads using the HG008 dataset.**

| **Baseline** | **Method** | **Variant Type** | **Total# in Baseline** | **Total# in Case** | **TP*** | **FP*** | **FN** | **Recall (%)** | **Precision (%)** | **F1-score (%)** |
| --- | --- | --- | --- | --- | --- | --- | --- | --- | --- | --- |
| SR_Consensus | nf-core/  pacsomatic | SNV | 9608 | 9952 | 9182 | 770 | 426 | 95.57 | 92.26 | 93.89 |
|  |  | Indel | 649 | 726 | 624 | 102 | 25 | 96.15 | 85.95 | 90.76 |
|  |  | SNV + Indel | 10257 | 10678 | 9806 | 872 | 451 | 95.6 | 91.83 | 93.68 |

* TP, true positives (variants present in both baseline and test set); FP, false positives (variants called by nf-core/pacsomatic but absent from baseline); FN, false negatives (variants in baseline but missed by nf-core/pacsomatic)

## **Supplementary Table 2. Comparison of structural variants calls of the nf-core/pacsomatic vs. the reference calls from GIAB**

| **SV Type** | **True Positives** | **False Positives** | **False Negatives** | **Precision (%)** | **Recall (%)** | **F1-Score (%)** |
| --- | --- | --- | --- | --- | --- | --- |
| DEL | 65 | 56 | 10 | 53.7 | 86.7 | 66.3 |
| INS | 26 | 48 | 20 | 35.1 | 56.5 | 43.3 |
| DUP | 40 | 20 | 3 | 66.7 | 93 | 77.7 |
| INV | 4 | 24 | 15 | 14.3 | 21.1 | 17 |
| BND | 30 | 29 | 30 | 50.8 | 50 | 50.4 |
| Overall | 165 | 177 | 78 | 48.2 | 67.9 | 56.4 |

**Supplementary Table 3. Benchmark evaluation of somatic caller of ClairS and Deepsomatic against SEQC somatic reference**

| **Caller** | **Variant Type** | **Truth_total** | **TP** | **FP** | **FN** | Recall(%) | **Precision(%)** | **F1(%)** |
| --- | --- | --- | --- | --- | --- | --- | --- | --- |
| ClairS | SNV | 39447 | 35670 | 2361 | 3777 | 0.9043 | 0.9379 | 0.9208 |
| ClairS | INDEL | 1625 | 1259 | 2932 | 366 | 0.7748 | 0.3004 | 0.4329 |
| ClairS | ALL | 41072 | 36929 | 5293 | 4143 | 0.8991 | 0.8746 | 0.8867 |
| DeepSomatic | SNV | 39447 | 37305 | 2038 | 2142 | 0.9457 | 0.9482 | 0.9469 |
| DeepSomatic | INDEL | 1625 | 1298 | 321 | 327 | 0.7988 | 0.8017 | 0.8002 |
| DeepSomatic | ALL | 41072 | 38603 | 2359 | 2469 | 0.9399 | 0.9424 | 0.9411 |

**Supplementary Table 4. Benchmark evaluation of germline caller of Clair3 and Deepvariant for normal (HCC1395-BL) and tumor (HCC1395) sample**

| Sample | Variant Type | Common | Clair3-unique | DeepVariant-unique | Clair3_total | DeepVariant_total | Jaccard |
| --- | --- | --- | --- | --- | --- | --- | --- |
| HCC1395-BL | SNV | 2,829,726 | 2,904 | 1,354 | 2,832,630 | 2,831,080 | 0.9985 |
| HCC1395-BL | INDEL | 174,972 | 695 | 2,162 | 175,667 | 177,134 | 0.9839 |
| HCC1395-BL | ALL | 3,004,698 | 3,599 | 3,516 | 3,008,297 | 3,008,214 | 0.9976 |
| HCC1395 | SNV | 2,328,647 | 17,240 | 7,203 | 2,345,887 | 2,335,850 | 0.9896 |
| HCC1395 | INDEL | 145,024 | 1,439 | 2,704 | 146,463 | 147,728 | 0.9722 |
| HCC1395 | ALL | 2,473,671 | 18,679 | 9,907 | 2,492,350 | 2,483,578 | 0.9886 |

## **Supplementary Figure 1. Comparison of variant detection between nf-core/pacsomatic and short-read callers.** The figure illustrates read alignments for two genomic examples, with short-read data displayed in the top two panels and long-read data in the bottom two. Left (chr4:19142): A 14bp deletion near the telomeric region was identified exclusively by the long-read workflow. Short-read sequencing failed to capture this variant because the limited read length resulted in low mapping quality, preventing reliable detection. Right (chr5:165755085): A 28bp deletion was missed by short-read sequencing due to high sequence complexity and associated mapping challenges in this region, whereas long reads successfully resolved the site.

## **Supplementary Figure 2. Overlapping of the called SVs from GIAB reference call set and nf-core/pacsomatic.** Among the 165 concordant SVs, 10 variants (6.1%) overlapped with pancreatic cancer genes, affecting seven distinct loci: CDKN2A, SMAD4, ATM, ARID1A, TGFBR2, GNAS, and CCND1. Notably, a 19.8 kb deletion at chr9:21,952,492-21,972,343 was detected within the CDKN2A tumor suppressor locus, representing a high-confidence somatic event validated by both pipelines. nf-core/pacsomatic identified 3 additional high-confidence SVs overlapping with cancer genes: a KRAS duplication (DV=64, VAF=35%) and two APC inversions (DV=55-58, VAF=65-66%), all validated by manual IGV inspection.

## **Supplementary Figure** **3. Venn diagram illustration of somatic variants (SNV and INDEL) called by ClairS and DeepSomatic and labeled with PASS at the High Confidence (HC) regions.**

**A**

**B**

**Supplementary Figure 4. VAF distribution of TP, FP, and FN by ClairS and DeepSomatic.** A) SNV; B) INDEL.

**D**

**C**

**A**

**B**

**Supplementary Figure 5. INDEL length distribution by ClairS and DeepSomatic at** A) high Confidence regions, and different VAF regions; B)different set as TP, FP, and FN.

**skip_deepvariant=false**

## **Supplementary Figure 6. The workflow of nf-core/pacsomatic when DeepVariant is selected as the germline short variant caller.**

**A**

**C**

**D**

**B**

## **Supplementary Figure 7.** **Venn diagram illustration of short germline variants called by Clair3 and DeepVariant and labeled with PASS.** A) SNV for Normal -Sample HCC1395-BL; B) INDEL for Normal- Sample HCC1395-BL; C) SNV for Tumor- Sample HCC1395; and D) INDEL for Tumor- Sample HCC1395.

## **Supplementary Figure 8. Comparison evaluation of the detected differential methylation regions due to the upstream germline caller Clair3 and DeepVariant.** A) Venn diagram of DMRs, B) Consistence plot of the methylation values.

**A**

**B**

**C**

**D**

## **Supplementary Figure 9. Computation efficiency and resource requirement of nf-core/pacsomatic running HG008 tumor/normal pairs.** nf-core/pacsomatic documents computational efficiency and resource demands within the pipeline_info directory. The generated HTML report includes visualized distributions for (**A**) CPU, (**B**) memory, (**C)** job duration, and (**D**) I/O, providing a comprehensive overview of the resources utilized by each process.
